# *Lama1 *mutations lead to vitreoretinal blood vessel formation, persistence of fetal vasculature, and epiretinal membrane formation in mice

**DOI:** 10.1186/1471-213X-11-60

**Published:** 2011-10-14

**Authors:** Malia M Edwards, D Scott McLeod, Rhonda Grebe, Céline Heng, Olivier Lefebvre, Gerard A Lutty

**Affiliations:** 1 The Wilmer Eye Institute, 400 N. Broadway, Baltimore, MD 21287, USA; 2 INSERM U682, Strasbourg, France

## Abstract

**Background:**

Valuable insights into the complex process of retinal vascular development can be gained using models with abnormal retinal vasculature. Two such models are the recently described mouse lines with mutations in *Lama1*, an important component of the retinal internal limiting membrane (ILM). These mutants have a persistence of the fetal vasculature of vitreous (FVV) but lack a primary retinal vascular plexus. The present study provides a detailed analysis of astrocyte and vascular development in these *Lama1 *mutants.

**Results:**

Although astrocytes and blood vessels initially migrate into *Lama1 *mutant retinas, both traverse the peripapillary ILM into the vitreous by P3. Once in the vitreous, blood vessels anastomose with vessels of the vasa hyaloidea propria, part of the FVV, and eventually re-enter the retina where they dive to form the inner and outer retinal capillary networks. Astrocytes continue proliferating within the vitreous to form a dense mesh that resembles epiretinal membranes associated with persistent fetal vasculature and proliferative vitreoretinopathy.

**Conclusions:**

*Lama1 *and a fully intact ILM are required for normal retinal vascular development. Mutations in *Lama1 *allow developing retinal vessels to enter the vitreous where they anastomose with vessels of the hyaloid system which persist and expand. Together, these vessels branch into the retina to form fairly normal inner retinal vascular capillary plexi. The *Lama1 *mutants described in this report are potential models for studying the human conditions persistent fetal vasculature and proliferative vitreoretinopathy.

## Background

The retinal vasculature, which nourishes the inner retina, consists of three plexi: the superficial in the nerve fiber layer, the intermediate in the inner plexiform layer, and the deep in the outer plexiform layer. The photoreceptors in the outer retina are maintained by the choroidal vessels. Retinal vessels develop in man during fetal development [1] but in mice during the first 3 post-natal weeks [2]. Prior to the formation of retinal vessels, the lens and developing inner retina are nourished by the hyaloid vasculature. This three component vasculature (hyaloid artery, vasa hyaloidea propria, and tunica vasculosa lentis) lies in the vitreous, a gel-like structure separating the retina and the lens. The hyaloid vessels regress as retinal vessels form and are gone in humans by birth and by three weeks in the mouse. The portion of the hyaloid vasculature closest to the retina is the vasa hyaloidea propria (VHP) which is also the last to regress.

**Figure 1:**
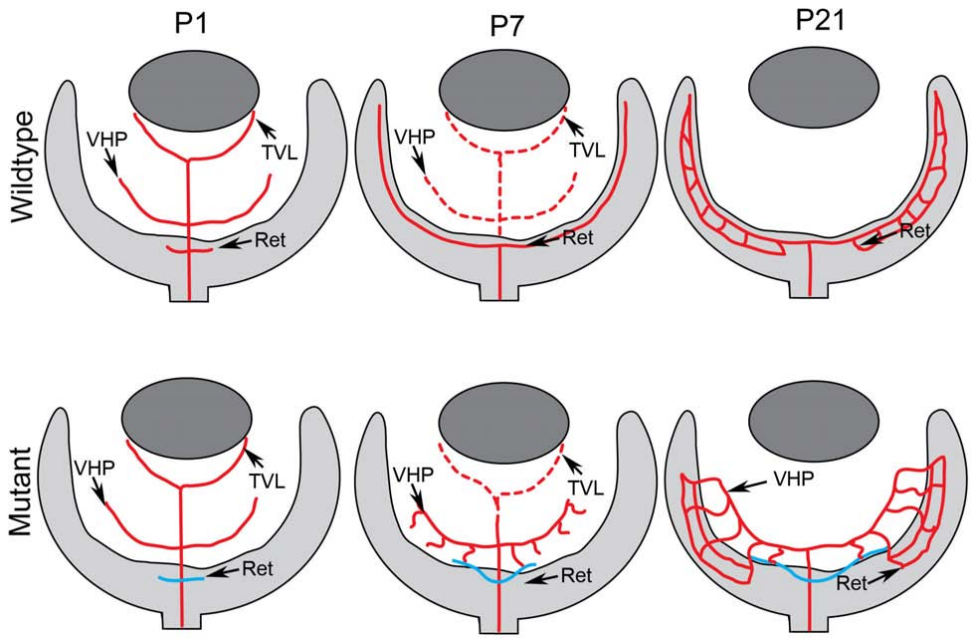
**A schematic of retinal vascular development**. The key stages in vascular development of the normal mouse retina (top) and the *Lama1^nmf223 ^*mutant retina (bottom) are represented in this diagram. Solid red lines depict blood vessels while blue lines indicate vessels which originate in the *Lama1 *mutant retinas but traverse the ILM into the vitreous beyond the peripapillary region. Broken red lines depict the regressing hyaloid vasculature. At P1, vessels have begun forming in both the control and *Lama1 *mutant retinas. The fetal vessels of vitreous (FVV), which includes the tunica TVL and the VHP, are regressing in the control by P7 and the primary retinal plexus is complete, while in the *Lama1 *mutants, the FVV have undergone some regression but those closest to the retina have proliferated. Retinal vessels (blue) have traversed the ILM to anastomose with the VHP vessels in P7 *Lama1 *mutants. The retinal vasculature is complete at P21 in the control retina and no hyaloid vessels remain in the vitreous. The FVV in the *Lama1 *retina at P21 persist and have proliferated and formed a retinal vasculature. (TVL-Tunica vasculosa lentis; VHP-vasa hyaloidea propria).

Although occurring prior to birth in the human and post-natally in the mouse, the retinal vasculature develops in a similar pattern in these species. The primary vascular plexus forms first and, once complete, branching vessels dive into the inner retina to form the deep plexus and then the intermediate plexus (Figure 1). The stimuli guiding the formation of retinal vessels, has yet to be confirmed. While in the mouse retina, endothelial cells migrate and proliferate across the retina guided by an astrocyte template [2,3], other guidance systems determine the pattern in dog [4] and man [5-7]. Furthermore, many studies have pointed towards VEGF as a key regulator in vascular development [8,9], but it has recently been demonstrated that retinal vessels form normally without astrocyte-derived VEGF [10]. Together, these data suggest that other proteins and, potentially, cells other than astrocytes guide endothelial cell migration in forming the superficial retinal vasculature.

The proximity of the internal limiting membrane (ILM) to retinal astrocytes and the superficial vasculature suggests that this structure may be important for development of retinal blood vessels. Indeed, several mouse mutants with disruptions to the ILM have an abnormal retinal vasculature [11-14]. Laminin α1 is a primary component of laminin 111, which is believed to be important for basement membrane formation [15-18]. In addition, this laminin chain links the ILM to receptors, such as integrins and dystroglycan [19], which are found on Müller cells in the retina [20,21]. Retinal vascular defects have recently been described in two mouse lines with mutations in *Lama1*, the gene which encodes laminin α1 [11]. While the vascular defects are similar, the two mutants differ in their generation and effect on laminin α1, one being a recessive point mutation (*Lama1^nmf223^*) and the other being a conditional knockout that leads to the complete loss of *Lama1 (Lama1^tm1.Olf^*, herein referred to as *Lama1^Δ^) *but bypasses the embryonic lethality normally associated with laminin α1 deletion. In the *Lama1^nmf223 ^*mice, a chemically-induced mutation causes the replacement of a tyrosine with a cysteine at amino acid 265 (Y265C) in the N-terminal domain, which includes sites for receptors binding as well as polymerization [11]. Here forward, *Lama1^nmf223 ^*and *Lama1^Δ ^*refer to mice homozygous for the respective mutations.

The present study investigates the development of retinal vessels in the previously described *Lama1 *mutants. Focus was placed on the *Lama1^nmf223 ^*because a random point mutation has greater potential to be associated with human disease than the complete deletion of *Lama1*, which is embryonic lethal in mice [15,22] and likely to cause lethality in humans as well. The observation that a point mutation in this large gene causes retinal disease in mice suggests that it may also do so in humans. Transmission electron microscopy (TEM) was used for ultrastructural analysis of the ILM, glial cells, and blood vessels in these mice. Finally, epiretinal membranes are described in both of these mutants that have characteristics similar to human persistent fetal vasculature (PFV) and proliferative vitreoretinopathy (PVR).

## Results

### Retinal vessels form in the Lama1^nmf223 ^retina at P1

**Figure 2:**
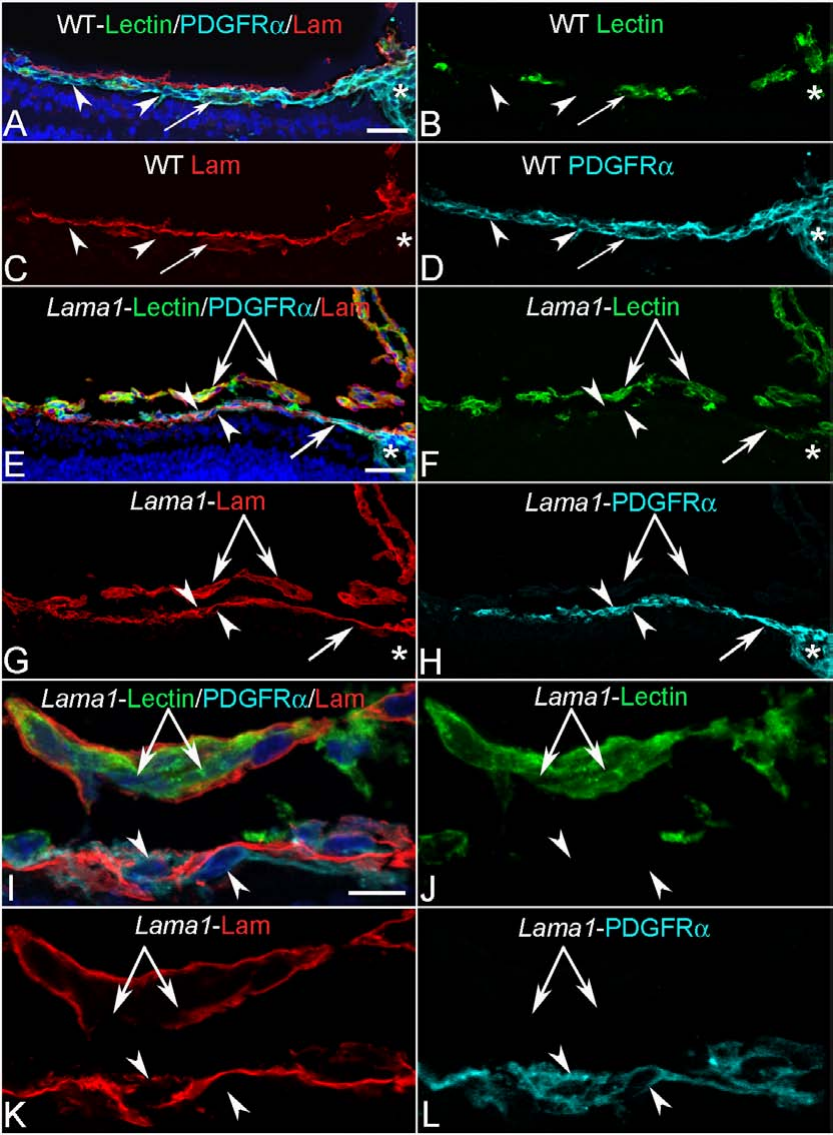
**Cross sections of whole eyes from P1 mice labeled with anti-PDGFRα (light blue), GS isolectin (green), anti-pan laminin (red), and DAPI (blue)**. Shown are both merged images (A, E, I) and individual labels. In the WT retina (A-D), astrocytes (arrowheads) and blood vessels (arrows) extended radially from the optic nerve head (asterisks) but, with the exception of a few astrocytes on the hyaloid artery, were present under the laminin-positive ILM (C). In the *Lama1^nmf223 ^*mice (E-H), astrocytes began migrating across the retina under the laminin-positive ILM (upward-facing arrowhead) but exited the retina once outside the peripapillary region (downward facing arrowhead). Blood vessels (arrow) were also present in the peripapillary retina. Individual optical slices taken from high magnification confocal Z stack images of the same area (I-L) revealed openings or breaks in the ILM through which astrocytes migrated. Astrocytes were seen in the retina (upward-facing arrowheads) and on the vitreal surface of the retina (downward-facing arrowheads) near a break in the ILM. The double arrow indicates the VHP, which in this area is devoid of astrocyte ensheathment. Scale bars indicate (A-H: 50 μm; I-L: 10 μm).

Blood vessel and astrocyte development was first investigated in cross sections by labeling with anti-pan laminin, anti-platelet-derived growth factor receptor α (PDGFRα) and GS isolectin at postnatal day (P) 1 when the primary retinal vasculature has just begun to form. In the wild type (WT) retina, astrocytes and blood vessels extended from the optic nerve head under the laminin-positive ILM (Figure 2A-D). Although some astrocytes were also observed along the base of the hyaloid artery, the vast majority of these glial cells were observed under the ILM. No astrocytes were observed in the vitreous away from the hyaloid artery. In the *Lama1^nmf223 ^*sections, blood vessels and PDGFRα-positive astrocytes were observed on the retinal side of the ILM in the peripapillary region, the area surrounding the optic nerve head (Figure 2E-H). Adjacent to this region, however, astrocytes were observed traversing the ILM into the vitreous (Figure 2E-L). In addition, some sections demonstrated astrocytes migrating into the vitreous along the hyaloid vessels (shown later at P3). Sequential confocal Z stacks were used to evaluate these sections and revealed openings in the ILM through which astrocytes entered the vitreous (Figure 2I-L). Peripheral to these "break" points, astrocytes were observed in a linear arrangement on the vitreal aspect of the ILM and only an occasional astrocyte was observed in the retina (Figure 2I, K). Unlike those in the retina, vitreal astrocytes expressed laminin.

**Figure 3:**
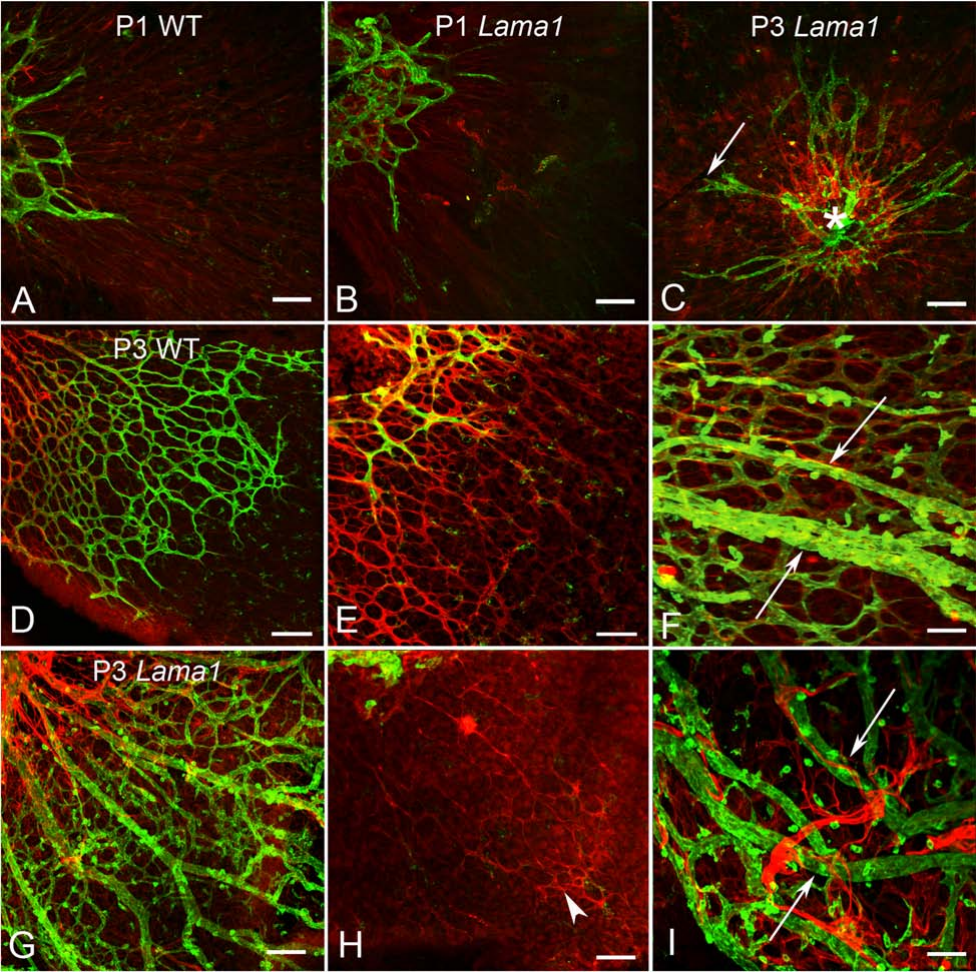
**P1 and P3 flatmount retinas labeled with anti-GFAP (red) and GS isolectin (green)**. At P1, removal of the vitreous revealed an apron of vessels around the optic nerve head behind an astrocyte template in both the WT (A) and the *Lama1^nmf223 ^*retinas (B). A similar image was observed in the *Lama1^nmf223 ^*retina at P3 when the vitreous was removed (C). The WT vasculature (arrow) extended into the mid retina at P3 (D). Higher magnification more clearly demonstrated the endothelial filopodia following the GFAP-positive astrocyte template in the WT retina (E). When imaged with the vitreous intact, it is clear that astrocytes ensheath the retinal vessels but not the hyaloid (arrow) in the WT (F). By contrast, imaging of the *Lama1^nmf223 ^*retina with the vitreous still intact, demonstrated the migration of astrocytes into the vitreous (G). The hyaloid vessels are proliferating at this stage rather than regressing. Removal of the vitreous demonstrated that very few astrocytes (arrowhead) remained in the retina beyond the peripapillary region (H). Astrocytes could be seen ensheathing the vitreal blood vessels (arrows) in the *Lama1^nmf223 ^*retinas (I). A capillary network had started to form in the vitreous of the *Lama1^nmf223 ^*mutants (G). Scale bars indicate A- D: 100 μm; D-I: 50 μm).

Flatmount WT retinas were labeled with fluorescein isothiocyanate conjugated *Griffonia simplicifolia *isolectin B4 (GS isolectin) to visualize blood vessels and anti-glial fibrillary acidic protein (GFAP) to visualize astrocytes. This labeling revealed a vascular apron surrounding the optic nerve head with endothelial cell filopodia extending out over an astrocyte template (Figure 3A). When present, hyaloid vessels were easily distinguished from those in the retina by the plane of focus and their GS isolectin intensity. A similar vascular apron was observed in *Lama1^nmf223 ^*mice (Figure 3B). In addition, a number of individual GS isolectin positive cells were observed in both the WT and *Lama1^nmf223 ^*retinas. Some of these cells are microglia, which GS isolectin also labels, as they also were positive for IBA-1 (data not shown).

**Figure 4:**
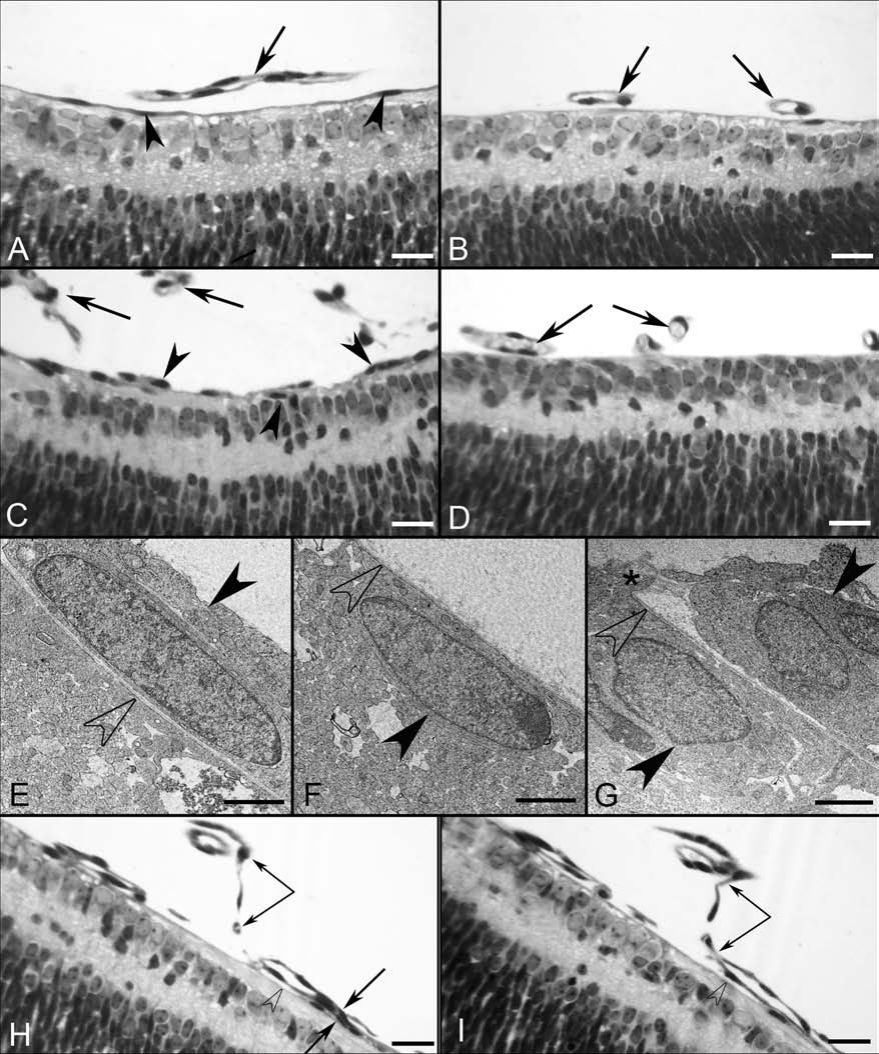
**Plastic sections of P1 and P3 WT and *Lama1^nmf223 ^*eyes**. JB-4 sections of P1 WT peripapillary (A) and midperiphery (B) retina show astrocytes (arrowheads) in retina and VHP (arrows) in vitreous. Sections from P1 *Lama1^nmf223 ^*retinas stained with hematoxylin and PAS (C, D) showed the VHP in vitreous (arrows) as well as astrocytes in the retina (upward arrowhead) and on the vitreal surface of the ILM (downward arrowhead). Astrocytes were only observed in the retina within the peripapillary region (C). Beyond this point, these glial cells (downward arrowheads) were found on the vitreal aspect of the ILM (C). Astrocytes were not observed in the retina peripheral to traversing the ILM (D). TEM analysis of P1 *Lama1^nmf223 ^*retina (E-G) showed the ILM (open arrowheads) and astrocytes on both the vitreal (solid downward arrowheads) and retinal sides of the ILM (solid upward arrowhead). The ILM is incomplete in this area having only a single lamina. Müller cell endfeet (asterisk) can also be seen protruding through the ILM in an area where this structure is otherwise complete (G). JB-4 analysis of P3 whole eyes demonstrated the migration of retinal blood vessels (arrows) from the retina into the vitreous (H). In a neighboring section, the retinal vessels and VHP (paired arrows) can be seen anastomosing in the vitreous (I). Open arrowheads indicate the ILM. Scale bars indicate (A-D, E-F: 20 μm; C, D: 1 μm, G: 2 μm).

JB-4 sections taken from WT eyes that were stained with periodic acid-Schiff (PAS) and hematoxylin revealed that all astrocytes were under the fully intact ILM (Figure 4A, B). JB-4 sections also demonstrated astrocytes leaving the retina in the peripapillary region of *Lama1^nmf223 ^*mice (Figure 4C). Peripheral to this point, astrocytes were not observed in the retina (Figure 4D). Ultrastructure analysis with TEM confirmed that astrocytes migrate into the vitreous through openings/breaks in the ILM. Müller cell processes were also seen extending into the vitreous at these break points (Figure 4G). Although they express GFAP, the astrocytes in the vitreous have few, if any, well-defined intermediate filaments at this stage, demonstrating that they are still maturing (Figure 4E). The migration of astrocytes into the vitreous near the peripapillary region and on the hyaloid artery was observed consistently in all eyes examined with the different techniques.

### Lama1^nmf223 ^retinal vessels migrate into the vitreous by P3

**Figure 5:**
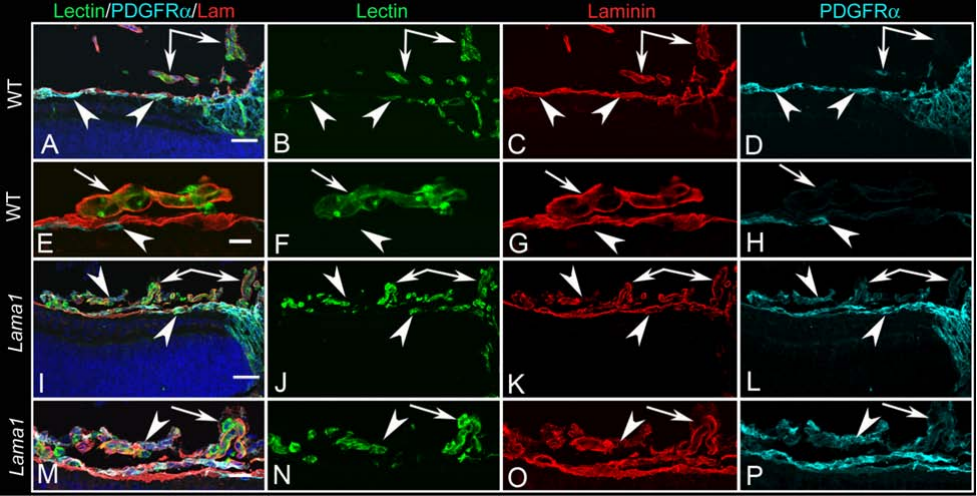
**Cross sections of whole eyes from P3 mice labeled with anti-PDGFRα (light blue), GS isolectin (green), anti-pan laminin (red), and DAPI (blue)**. Shown are both merged images (A, E, I, M) and individual labeling. In the WT retina (A-D), astrocytes (upward arrowheads) and blood vessels extended out from the optic nerve head but were contained under the laminin-positive ILM. The VHP and hyaloid vessels within vitreous (paired arrows) were still present. Higher magnification images of the WT retina confirmed that, while there were a few astrocytes associated with the VHP (arrow) at the optic nerve head (D), most astrocytes (arrowheads) were found within the retina (E-H). In the P3 *Lama1^nmf223 ^*retina (I-L), the density of intraretinal astrocytes (upward arrowheads) was similar to that seen at P1 but there appeared to be more astrocytes within the vitreous (downward arrowheads) ensheathing the VHP and hyaloid vessels (paired arrows). Higher magnification images (M-P) demonstrated astrocytes (arrowheads) associating with VHP (arrow). As seen at P1, astrocytes within the vitreous were positive for anti-pan laminin (O). Scale bars indicate (A-D, I-L: 50 μm; E-H, M-P: 20 μm).

Examination of retinal flatmounts at P3 revealed the extension of blood vessels in the WT retina to the midperiphery behind a fully-formed astrocytic template (Figure 3D, E). The hyaloid vessels, which detached from the retina during dissection, were still present but were not ensheathed with retinal astrocytes (Figure 3F). Immunohistochemical labeling of cross sections demonstrated that retinal vessels and astrocytes were located on the retinal side of the ILM (Figure 5A-D). One or two astrocytes were observed along the hyaloid artery right at the optic nerve head (Figure 5D). Despite the close proximity of the VHP vessels to the ILM, they did not cross this barrier or make contact with retinal astrocytes in the WT eyes (Figure 5E-H). By contrast, the vessels in the *Lama1^nmf223 ^*retinas had not progressed from that seen at P1 in retinal flatmounts (Figure 3C). There were also less astrocytes in the mutant retina (Figure 3H) compared to the WT (Figure 3E). A number of GS isolectin-positive cells were, however, observed throughout both the WT (Figure 3D, E) and *Lama1^nmf223 ^*retinas (Figure 3C, G). These cells were more prominent in the avascular regions of both the WT and the *Lama1^nmf223 ^*retinas. Imaging of retinas with the vitreous intact demonstrated that most of the *Lama1^nmf223 ^*astrocytes had migrated into the vitreous where they invested the VHP (Figure 3G-I). Astrocytes were also observed forming thin glial bridges connecting blood vessels within the vitreous (Figure 3I). Capillary networks were seen branching from the *Lama1^nmf223 ^*VHP (Figure 3G). As was observed at P1, cross sectional analysis revealed astrocytes migrating from the retina into the vitreous both at the optic nerve head and through breaks in the ILM (Figure 5I-P). These astrocytes extended in a linear fashion along the vitreal aspect of the ILM. At this stage, astrocytes were clearly enveloping VHP vessels and forming a laminin-positive membrane-like structure. The retinal vessels observed in the peripapillary region at P1 had also migrated into the vitreous. JB-4 cross sections demonstrated that these vessels had begun to anastomose with vessels of the VHP (Figure 4H-I).

### Only intravitreal vessels are evident in Lama1^nmf223 ^mice at P7

**Figure 6:**
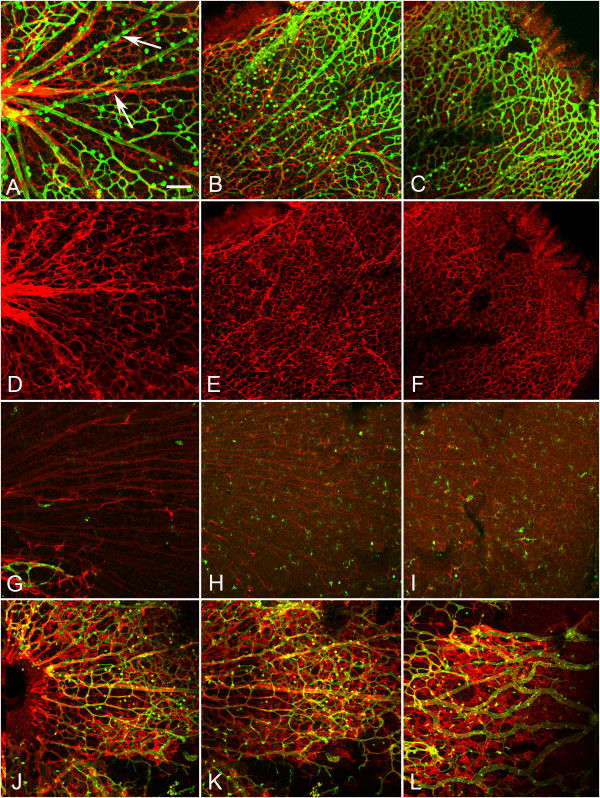
**P7 flatmount retinas labeled with anti-GFAP (red) and GS isolectin (green)**. A complete primary retinal vascular network and astrocyte template were observed in the WT mouse at P7 (A, D = peripapillary region, B, E = midperiphery C, F = far periphery). The GS isolectin positive cells in A are likely hyalocytes on the VHP (arrows). Remodeling of the retinal blood vessels had also already occurred in the control retina (A). The astrocyte template was more evident with the green channel turned off (D-F). By contrast, retinal vessels were observed only at the optic nerve head (bottom left) in the *Lama1^nmf223 ^*retina (G) and not in mid (H) or far periphery (I). Isolated GS isolectin positive cells could be seen across the retina. Astrocytes in the mutant retina (G-I) were reduced in number and did not have the honeycomb-like pattern observed in the WT (D-F). When the vitreous was left intact, a dense astrocyte mesh was observed along with a vitreal capillary network in all regions (J = peripapillary, K = mid retina, and L = peripheral retina). Scale bars indicate 100 μm.

**Figure 7:**
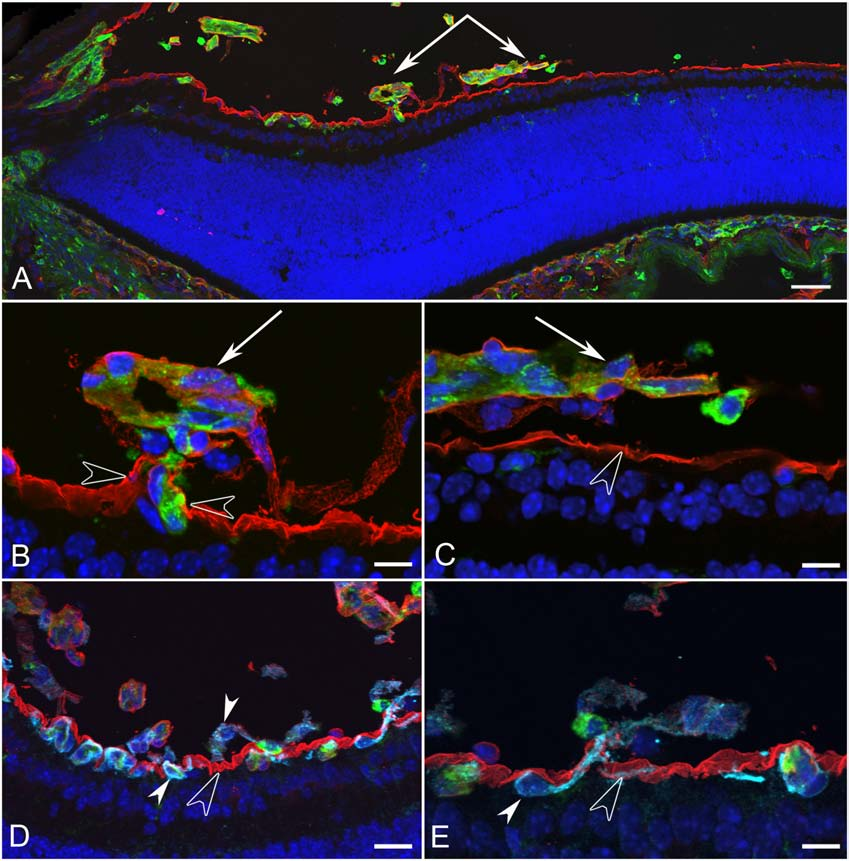
**Cross sections of whole eyes from *Lama1^nmf223 ^*P7 mice were labeled with GS isolectin (green), anti-pan laminin (red), and DAPI (blue)**. A panoramic composite of a representative *Lama1^nmf223 ^*retina demonstrated the migration of blood vessels out of the retina towards the VHP (paired arrows) and across the vitreal surface of the ILM (A). Higher magnification of this area (B, C) demonstrated the presence of a laminin-positive membrane-like structure surrounding the exiting blood vessels (arrows) and exiting through the ILM (opposing open arrowheads). Labeling of an adjacent section (D) with anti-PDGFRα (light blue) along with GS isolectin (green), anti-pan laminin (red), and DAPI (blue) showed astrocytes within retina (upward solid arrowhead) and on the vitreal side (downward arrowhead) of the ILM (open arrowhead). Higher magnification (E) showed an astrocyte (solid arrowhead) migrating from the retina to vitreous through the ILM (open arrowhead). Scale bars indicate (A: 50 μm; B, C, E: 10 μm; D: 20 μm).

At P7, the primary vascular plexus was complete in the WT retina with the exception of the extreme periphery in some retinas (Figure 6A-C). This vasculature included arteries and veins as well as the typical chicken wire-like capillary plexus. The astrocyte template was complete in WT mice at this stage with astrocytes ensheathing retinal vessels (Figure 6D-F). The hyaloid vasculature and VHP had begun regressing and detached easily from the retina during dissection. When present, these vessels had GS isolectin-positive cells, likely hyalocytes, on top of them (Figure 6A-C). By contrast, the *Lama1^nmf223 ^*retinal vasculature resembled that of the P3 retina, being limited to an apron in the peripapillary region. Retinal vessels were absent beyond this point and individual GS isolectin-positive cells were prominent (Figure 6G-I). There was a large reduction in the number of astrocytes within the mutant retina (Figure 6G-I) compared to the control (Figure 6D-F). While some peripheral retinal astrocytes formed a normal honeycomb-like pattern, most extended long thin processes across the retina. When the vitreous was left intact, a much different image was revealed (Figure 6J-L). A large number of astrocytes were observed in the vitreous where they ensheathed vessels and formed a dense membrane-like structure. The intravitreal capillary networks, which had begun forming at P3, were more extensive. In sections, anti-pan laminin and GS isolectin labeling demonstrated the peripapillary retinal vessels traversing the ILM into the vitreous where they continued to extend towards ora serrata (Figure 7A-C). A membrane-like laminin positive structure was forming in association with these vitreal vessels (Figure 7A, C). Peripheral to break points in the ILM, no intraretinal vessels were observed (Figure 7A). It is important to note that the laminin-positive ILM was also observed on either side of blood vessels traversing into the vitreous (Figure 7B). Labeling of adjacent sections with anti-PDGFRα in addition to anti-pan laminin and GS isolectin revealed that astrocytes crossed the ILM and were part of the vitreal membrane (Figure 7D-E). These astrocytes were positive for laminin. These astrocytes extended along the vitreal side of the ILM and towards ora serrata in a fashion similar to that observed within the inner retina of the WT mice (data now shown).

### Intravitreal vessels invade the *Lama1^nmf223 ^*retina by P10

**Figure 8:**
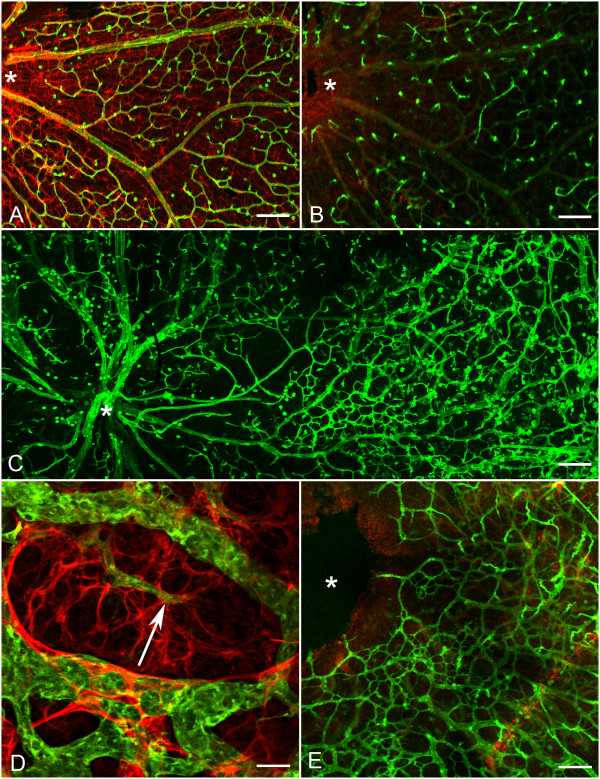
**P10 flatmount retinas labeled with anti-GFAP (red) and GS isolectin (green)**. A complete and remodeled primary or superficial vasculature was observed in the WT mouse (A) and the deep vascular plexus was forming at this stage (B). A panoramic image of the *Lama1^nmf223 ^*retina with only GS isolectin labeling (C) demonstrated the abnormal persistence of the VHP, growth of vessels into vitreous from the peripapillary retina and the formation of a complex capillary network within the retina. Double labeling with anti-GFAP and GS isolectin (D) showed continued association of astrocytes with the hyaloid vessels. There appeared to be two layers of astrocytes within the vitreous, one which ensheathed the persistent VHP and one below the vessels on the surface of the retina. Also evident is branching from the intravitreal vessels (arrow) into the retina (D) to form the intraretinal plexus (E). Asterisks mark the optic nerve head. Scale bars indicate (A-C: 100 μm; D, E: 50 μm).

**Figure 9:**
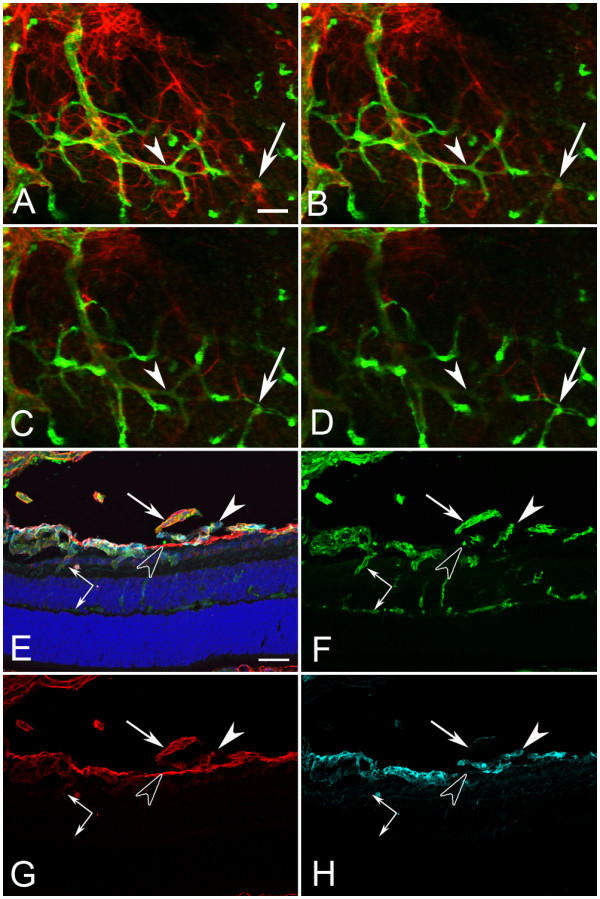
**The diving of vitreal vessels into the retina is evident with both flatmount and cross section analysis**. Sequential frames from a confocal Z stack image of a P10 flatmount *Lama1^nmf223 ^*retina labeled with GFAP and GS isolectin demonstrated the diving of vessels (arrows and arrowhead) into the retina (A-D). Cross sections from P10 *Lama1^nmf223 ^*eyes were labeled with anti-PDGFRα (light blue), GS isolectin (green), anti-pan laminin (red), and DAPI (blue) to support this observation. Vessels from the vitreous were branching into the retina at this stage, primarily in the inner plexiform layer, as the deep vascular plexus was forming (paired arrows) (E-H). Laminin-positive astrocytes (solid arrowhead) were also observed on the vitreal side of the ILM (open arrowhead) near the VHP (arrows) (E-H). Scale bars indicate (A-D: 20 μm; E-H: 50 μm).

**Figure 10:**
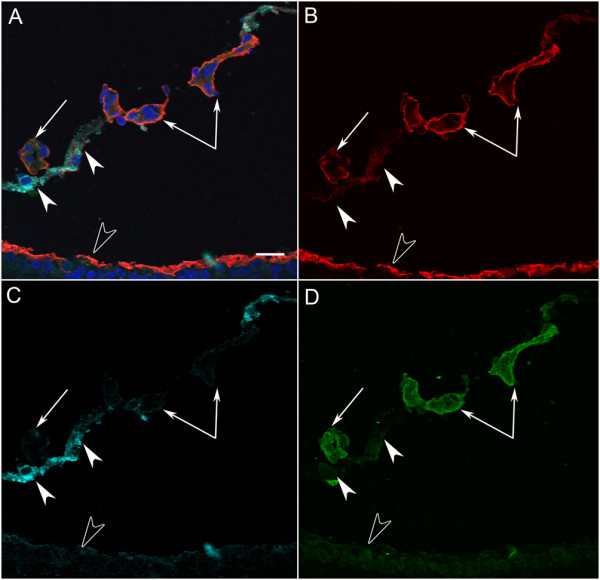
**A cross section from a P10 *Lama1^nmf223 ^*mouse labeled with GS isolectin (green), anti-pan laminin (red), anti-PDGFRα (light blue) and DAPI (blue)**. A PDGFRα-positive astrocyte bridge (solid arrowheads) (A, C), is attached to GS isolectin (green) positive intravitreal blood vessels (paired and single arrows) (A, D). The ILM (open arrowhead) and the basement membrane of the intravitreal vessels were laminin-positive (B). Scale bars indicate 20 μm.

**Figure 11:**
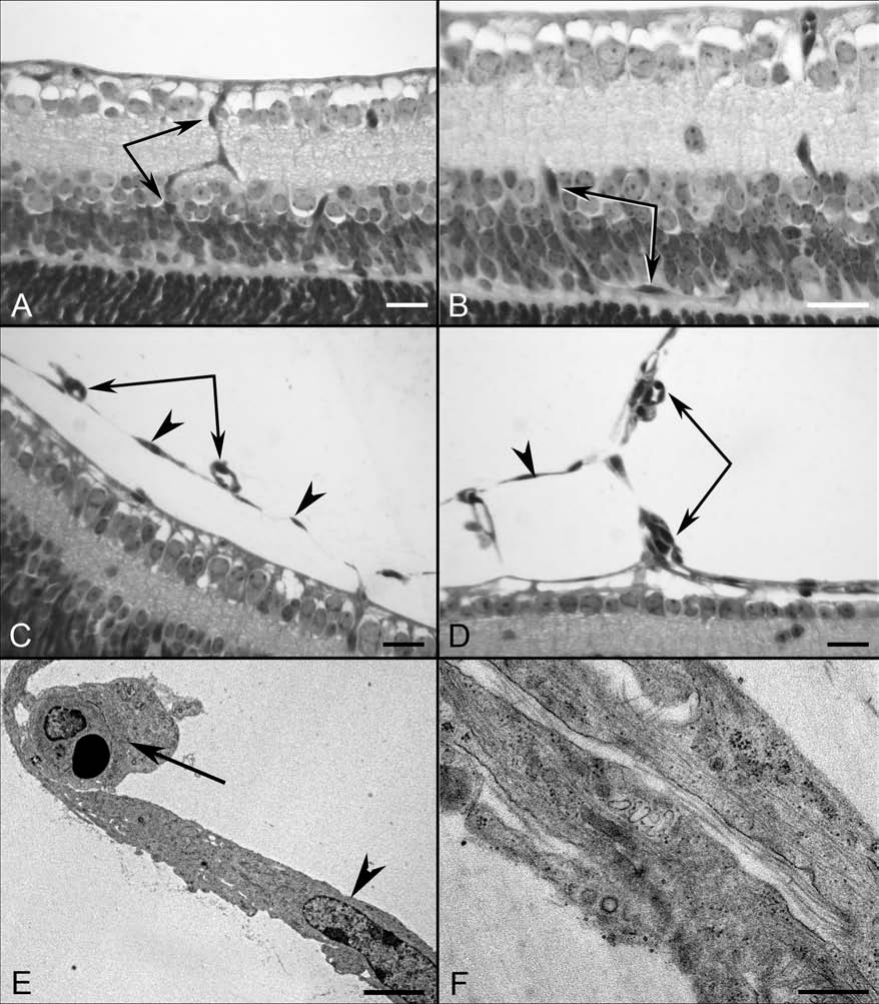
**Plastic sections of the P10 WT and *Lama1^nmf223 ^*eyes**. JB-4 cross sections revealed the diving of superficial retinal vessels (paired arrows) in the WT retina (A, B). In the *Lama1^nmf223 ^*retinas, astrocytes (arrowheads) and blood vessels (paired arrows) were in the vitreous and entering the retina (C, D). TEM confirms the presence of astrocytes (arrowhead) and blood vessels (arrow) in the vitreous (E) with intermediate filaments evident in astrocytes at high magnification (F). Scale bars indicate (A-D: 20 μm; E: 3 μm; F: 250 nm).

In the P10 WT retina, the superficial chicken wire capillary network was remodeled into a spoke-wheel-like system with an organized branching pattern (Figure 8A). Astrocytes had a distinct stellate morphology with thin processes that contacted and ensheathed retinal blood vessels (Figure 8A). Superficial vessels could be seen diving to form the deep capillary plexus (Figure 8B). GS isolectin labeling of the *Lama1^nmf223 ^*flatmount retinas demonstrated an extensive vascular network with an unusual branching pattern in the vitreous (Figure 8C). When the vitreous was left intact and retinas double-labeled with GS isolectin and anti-GFAP, the appearance was similar to that at P7 although the astrocyte "membrane" appeared to be denser. At this stage, branches from intravitreal vessels extended into the retina to form an intraretinal capillary plexus (Figure 8D, E). This abnormal branching was best appreciated with confocal Z stack images through the retina (Figure 9 A-D). Labeling of whole eye cross sections with anti-PDGFRα, anti-pan laminin, and GS isolectin more clearly demonstrated vessels within the vitreous and their branches that had invaded the inner and outer retina and formed a capillary network in the outer plexiform layer (Figure 9E-H). In addition to astrocytes migrating near the vitreoretinal surface, some had migrated further into the vitreous (Figure 10 A-D). Many of these astrocytes possessed long, thin processes and created glial bridges between intravitreal blood vessels. JB-4 sections of the WT retina revealed that astrocytes resided under the ILM and that the superficial vessels were diving into the deeper retinal layers (Figure 11A, B). In the *Lama1^nmf223 ^*P10 JB-4 sections, long thin processes from the glial bridges mentioned above as well as their frequent contact with blood vessels could be appreciated (Figure 11C). In some cases, they created tractional attachments with the vessels (Figure 11D). Astrocytes extended across the vitreal surface of the ILM to the far periphery. Some astrocytes appeared to have made contact with cells in the retina. Intravitreal vessels that had invaded the retina, extended into both the nerve fiber layer and into the deeper retina (Figure 9). Ultrastructural analysis demonstrated breaks in the ILM similar to those seen at P1 (data not shown). Astrocytes and blood vessels were closely associated in the vitreous (Figure 11E). Additionally, intermediate filaments were present in intravitreal astrocytes at this age (Figure 11F).

### Adult *Lama1^nmf223^*

**Figure 12:**
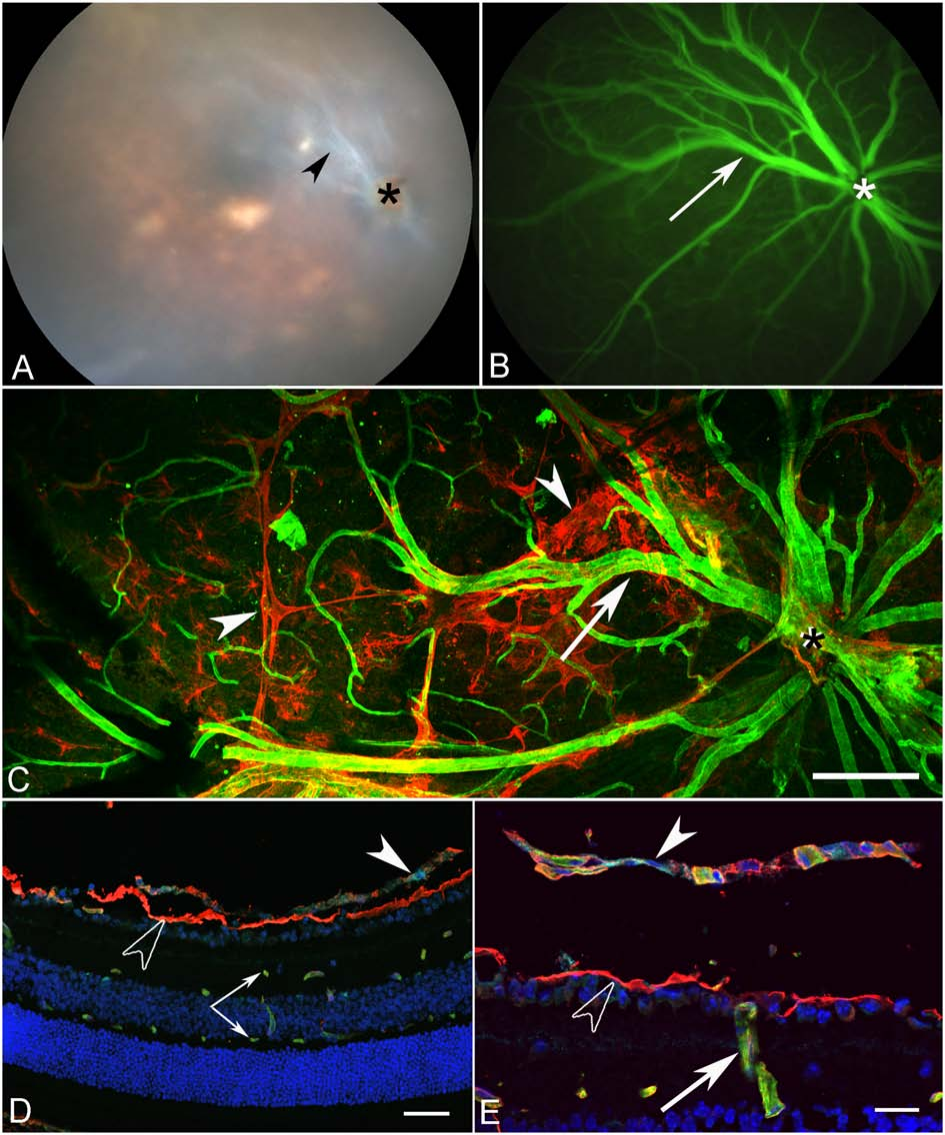
**The retinal phenotype in the adult *Lama1^nmf223 ^*mouse**. Fundus image (A) of the adult *Lama1^nmf223 ^*mouse reveals a membrane-like structure in the vitreous (arrowhead) surrounding persistent VHP near the optic nerve head (asterisk). Fluorescein angiography (B) confirms the presence of patent vessels (arrow) within the vitreous along with the inner retinal capillaries. Anti-GFAP (red) and GS isolectin (green) labeled flatmount retina (C) shows the extension of an astrocyte membrane (arrowheads) across the retina along the persistent VHP (arrow). Asterisks indicate the optic nerve head. Cross sections (D, E) labeled with anti-PDGFRα (light blue), anti-pan laminin (red) and GS isolectin (green), and DAPI (blue) further demonstrate the astrocytes in the vitreous (solid arrowhead) above the ILM (open arrowhead) and the presence of superficial and deep capillaries in the retina (paired arrows). Diving of vitreal vessels (arrow) into the retina was also observed (E). Scale bars indicate (C: 100 μm; D: 40 μm; E: 20 μm).

Fundus examination of four-week old *Lama1^nmf223 ^*mice demonstrated a white membrane-like structure surrounding hyaloid vessels in the vitreal plane of focus, (Figure 12A). The position of this membrane in relation to the vitreal blood vessels suggested it was the astrocytic membrane observed in flatmounts and sectioned retinas. Fluorescein angiography confirmed the presence of large patent vessels within the vitreous (Figure 12B). These vessels could be seen branching into the retina to form the inner retinal capillary system. Flatmount retinas labeled with anti-GFAP and GS isolectin confirmed the continuance of the astrocyte membrane in the vitreous of *Lama1^nmf223 ^*mutants (Figure 12C). Labeling with anti-PDGFRα, anti-pan laminin, and GS isolectin clearly showed a chain of PDGFRα-positive astrocytes across the vitreal surface of the ILM as well as the glial bridges between intravitreal blood vessels (Figure 12D, E). The vitreal vessels dove into the retina where they branched to form the inner and outer retinal capillary networks (Figure 12D, E).

### Abnormal retinal vascular development in the *Lama1^Δ ^mice *is similar to that in *Lama1^nmf223^*

**Figure 13:**
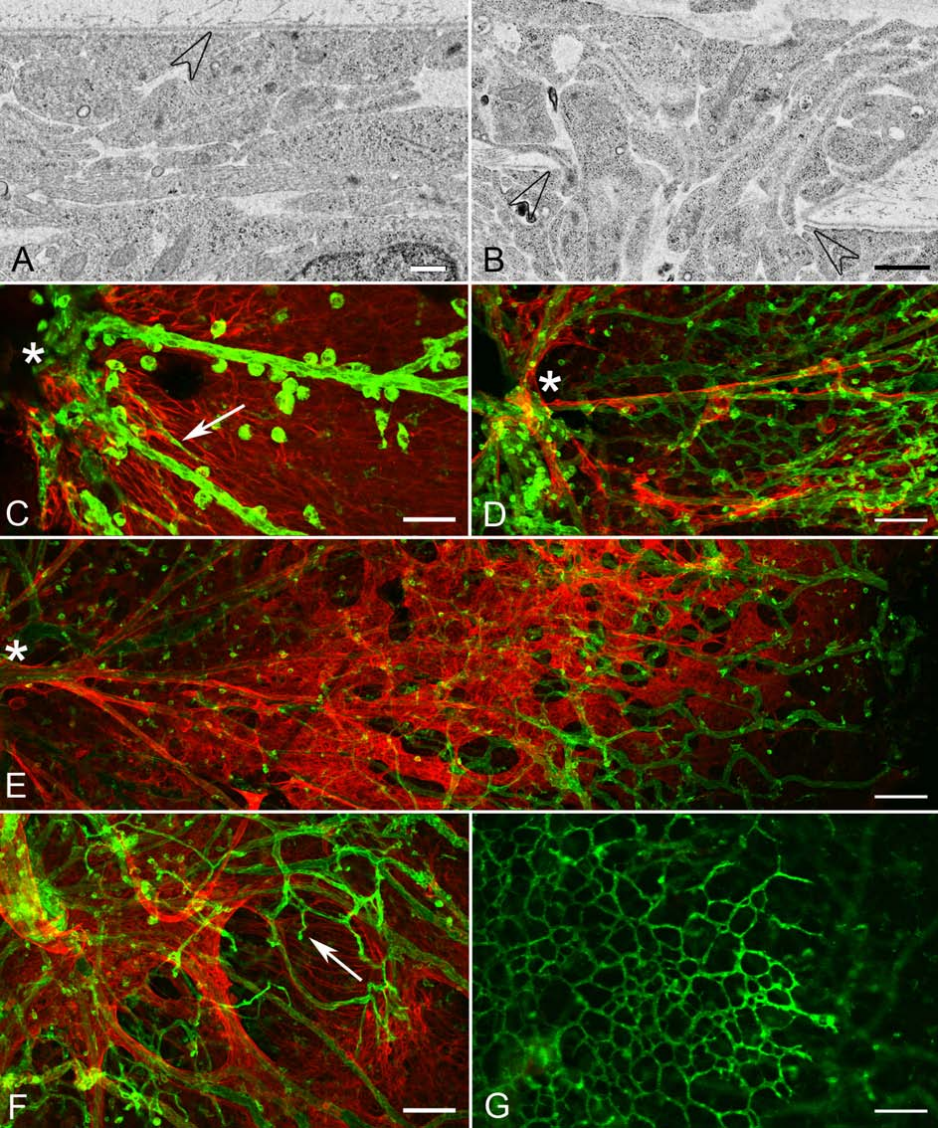
**Summary of vascular and astrocyte development in the *Lama1^Δ ^*retina TEM analysis of P1 WT retina demonstrates a double layered ILM (A)**. In contrast, the ILM in a *Lama1^Δ ^*mutant mouse contains frequent openings or breaks in this structure through which Müller cell processes extended into the vitreous (B). Open arrowheads point to the ILM. Examination of the *Lama1^Δ ^*mouse reveals a similar vascular development pattern similar to that seen in the *Lama1^nmf223 ^*mouse. (C) Anti-GFAP (red) and GS isolectin (green) labeling show the presence of a vascular apron (arrow) around the optic nerve head (asterisk) as well as large blood vessels of the hyaloid vasculature in the P1 *Lama1^Δ ^*mouse. At P7, astrocytes have entered the vitreous where they associate with hyaloid vessels (D). A dense astrocyte membrane and capillary network are observed in the vitreous across the entire retina at P10 (E). Diving vitreal vessels (arrows) can be also observed (F). A deep retinal plexus is forming from these diving vessels (G). Asterisks indicate the optic nerve head. Scale bars indicate (A: 500 nm; B: 2 μm; C, F: 50 μm; G, E, G: 100 μm).

Transmission electron microscopy revealed a double layered ILM which spanned the entire retina in the WT mouse at P1 (Figure 13A). By contrast, the P1 *Lama1^Δ ^*mice revealed a thin, fragmented ILM that contained frequent breaks through which astrocytes, Müller cells, and ganglion cells seemed to be protruding (Figure 13B). Astrocytes were observed on the vitreal side of the ILM (Additional File 1, Fig. S1). The vascular development in *Lama1^Δ ^*retinas was examined at key time points determined by investigation of the *Lama1^nmf223 ^*mutants: P1, P7, and P10. At P1, a small apron of vessels was evident at the optic nerve head, similar to that seen in the *Lama1^nmf223 ^*mutants (Figure 13C). When the vitreous was left intact, astrocytes were observed on the hyaloid vessels at P1. At P7, the GFAP and GS isolectin labeling produced images similar to those from the *Lama1^nmf223 ^*mice showing astrocytes associating with the VHP (Figure 13D) and no vessels within the retina (Additional File 2, Fig. S2). Labeling of the P10 retina revealed a dense astrocyte network present in the vitreous of the *Lama1^Δ ^*mice that was more dense than in the *Lama1^nmf223 ^*eye (Figure 13E) and intravitreal vessels extending into the retina (Figure 13F). In some retinas, GFAP-positive processes were also observed traversing the retina along with the blood vessels. The deep vasculature was developing from diving vitreal vessels at P10 (Figure 13G). In summary, the same changes occurred in the *Lama1^Δ ^*mice as in the *Lama1^nmf223 ^*mice. The progression, however, was slightly faster and the severity of the pathology was greater at least in terms of astrocyte coverage of the VHP.

## Discussion

The previous study reported that few, if any, retinal vessels developed in the *Lama1 *mutants and that these were replaced by persistent intravitreal vessels which penetrate the retina to form the intraretinal capillary networks [11]. Using confocal microscopy, additional immunohistochemical markers, cross sectional analysis, and TEM, the present study redefines the development of retinal vessels and astrocytes in the *Lama1 *mutants. In addition, the previously described "vitreal fibroplasia" is identified as a vitreal membrane containing laminin-positive astrocytes, similar to human persistent fetal vasculature.

The ILM in the *Lama1^nmf223 ^*retina is normal in appearance throughout much of the retina but has small frequent breaks. By contrast, the *Lama1^Δ ^*mice have a very thin ILM with a diffuse, nonlinear appearance throughout much of the retina. In addition, breaks in the *Lama1^Δ ^*retina generally extended over larger areas of the retina. In many areas, the ILM was lacking completely. These findings are not surprising because an ILM lacking laminin α1 would likely not be bound well to the retina as this protein contains the binding sites for receptors on Müller cell endfeet [20,21]. The preservation of the ILM in some areas suggests that other alpha chains present in the ILM, such as α5 [23], assist in binding this structure to the retina. Despite the differing ILM structure, the two *Lama1 *mutants have very similar abnormalities with regards to retinal vascular development.

Both mutants have a vascular apron around the optic nerve head at P1, suggesting that neither the Y265C mutation nor the complete loss of this protein affected endothelial cell differentiation and emigration from the optic nerve. Beginning around P3, however, it is evident that the retinal blood vessels cease to develop in the *Lama1^nmf223 ^*retina and have entered the vitreous where they remain until P7-P9. These vessels, which originate within the peripapillary retina grow along the retinal surface and eventually anastomose with those of the VHP. Together, these vessels form a dense vascular network in the vitreous that persists into adulthood and is ensheathed by astrocytes. These vitreal vessels sprout and invade the retina around P10 to form the retinal vascular capillary networks. Despite their abnormal origin, the *Lama1 *mutant retinal capillary networks at P10 (Figure 9E) are correctly placed within the retina. Therefore, the cues guiding these diving vessels are still present. It appears, however, as though the intermediate plexus forms early in the *Lama1^nmf223 ^*retina as vessels are evident in the inner plexiform layer at P10 compared to P14 in the WT retina.

The formation of the inner capillary networks further indicates that neither the Y265C point mutation in *Lama1 *nor the complete deletion of this protein alters the ability of endothelial cells to form blood vessels. Rather, astrocyte migration across the vitreal surface of the ILM precedes and likely causes the misguided migration of the blood vessels into the vitreous. In all retinas investigated at various ages, astrocytes were observed in the vitreous before retinal vessels traversed the ILM. In addition, only the VHP vessels were observed in the vitreous posterior to astrocytes on the vitreal aspect of the ILM. It is possible that if a normal template of astrocytes was present in the retina, the blood vessel development would also be normal. Therefore, identifying and neutralizing the factor(s) which stimulate astrocyte invasion into the vitreous may also alleviate the vascular defects in these mice. Most astrocytes from the *Lama1 *mutant retinas migrate towards and eventually envelop the VHP vessels. In addition, a subpopulation of these glial cells migrates from the optic nerve head directly into the vitreous along the hyaloid artery and its branches. These observations suggest that an attractant within the persistent and proliferating intravitreal vessels stimulates astrocyte migration. The previous report demonstrated that the tyrosine to cysteine mutation in the *Lama1^nmf223 ^*mice significantly decreases the binding ability of laminin α1 [11]. It is logical to hypothesize, therefore, that this mutation disrupts the strength by which laminin α1 and, consequently, the entire ILM, bind to receptors on Müller cell endfeet. As a result, the ILM may become less of a barrier for cells in the retina. Astrocytes may, therefore, respond to chemoattractants in the vitreous more easily than those in the WT retina. Astrocyte migration into the vitreous in response to these chemoattractants may create breaks in the ILM. This idea is supported by the fact that in most cases, both laminin immunohistochemistry and TEM show the ILM pushed inwardly toward the vitreous where the cells exit the retina rather than there being a clear opening in the ILM through which cells migrate (Figure 7B, 13B). Indeed, a number of proteins within the normal vitreous are known to stimulate astrocyte migration, including hyaluronic acid [24], endothelin-1 [25], TGF-beta, and PDGF-AA [8] . Among these, PDGF-AA is the best candidate as it is known to stimulate astrocyte migration and is believed to be generated by ganglion cells in the retina [8,26]. Furthermore, PDGF-AA overexpression by the lens stimulates astrocyte migration into the vitreous, resulting in a pattern similar to that observed in the *Lama1 *mutants [26].

Once within the vitreous, most astrocytes migrate along the vitreal surface of the ILM towards the ora serrata. This migration pattern is similar to that seen within WT retinas with the exception that it occurs on the vitreal side of the ILM. This observation indicates that the migratory cues normally present inside the retina are on the vitreal surface of the ILM in *Lama1 *mutants. It could also be hypothesized that proteins constituting the ILM act as guidance cues and astrocytes in the mutant retina respond to these but remain on the vitreal side once they have traversed the ILM. Yet another theory is that Müller cells, the other retinal glial cell, guide astrocyte migration. Although Müller cells may not be fully differentiated, these glial cells are in place prior to astrocyte migration into and across the retina. Furthermore, Müller cell processes in both *Lama1 *mutants extend into the vitreous where chemoattractants they produce could spill out. In addition, some Müller cell endfeet are properly positioned, potentially contributing to frequent contact between vitreal astrocytes and the retina. Further investigation is required to identify the stimuli that guide astrocytes across the retina and, perhaps more importantly, what cell type(s) provide these stimuli.

The association of astrocytes with hyaloid vessels occurs in other rodent mutants with persistent fetal vasculature, including *Collagen 15a1/18a1 *double knockouts [13], *LIF *transgenics [27], and the Nuc1 rat [28]. The question arises then, do astrocytes associate with vitreal vessels as a consequence of their failure to regress or is the astrocyte association preventing their regression by stabilizing the vasculature? In the case of the *Lama1 *mutants, the astrocytes migrate into the vitreous and associate with hyaloid and VHP by P3, before VHP regression has normally begun. This suggests that the astrocyte ensheathment of vitreal blood vessels subsequently inhibits the vessel regression and may even stimulate their proliferation.

Astrocyte ensheathment of the fetal vasculature in vitreous also occurs in the human disease PFV [29,30]. PFV can be a blinding disorder because the retinal vasculature is incomplete and the persistent FVV and membrane pulls on the retina causing detachment [31]. Preretinal glial membranes are often found in patients with PFV and likely contribute to retinal detachment [29,31,32]. It is not uncommon for patients with PFV to develop cataracts and glaucoma as well [31,33]. Few animal models exist for this condition and treatment involves surgery. The *Lama1 *mutants described herein have two hallmark features of this disease: persistence of the fetal vasculature and preretinal glial membranes, making them potential models for studying this disease.

The membranes formed by astrocytes in *Lama1 *mutants also resemble epiretinal membranes seen in patients with proliferative vitreoretinopathy (PVR), a complication of retinal detachment in which retinal cells and macrophages enter the vitreous. The ultrastructure of the glial-containing membrane in *Lama1 *mutants is similar to that described for epiretinal membranes [34,35]. In some areas, astrocytes extend thin, delicate processes across the vitreoretinal surface and into the vitreous. These created structures similar to those which Foos called glial bridges [34]. In others areas, numerous astrocytes overlap one another to form a dense mesh-like structure. While the glial membranes in *Lama1 *mutants contain mainly tightly associated astrocytes, frequent irregularly large openings are also observed (Figure 13E). The astrocytes associated with this membrane make laminin, suggesting they may also produce other extracellular matrix proteins, a key feature in PVR. Current animals models for PVR are invasive, either injection of fibroblasts into vitreous or surgical disruption of the ILM to stimulate membrane formation [36].

The *Lama1 *mutants described herein provide novel, genetic models for studying PVR and PFV. The production and expression of laminin by vitreal astrocytes in *Lama1^nmf223 ^*mutants but not those in the retina indicates that they could contribute to the generation, expansion, and stabilization of the preretinal membranes. Astrocyte expression of laminin α1 and γ1 has been observed in association with glial activation during CNS disease [37,38]. Furthermore, activated astrocytes express and secrete many cytokines which could alter the development of retinal vessels and promote membrane formation. A better understanding of how these vitreal membranes develop could help stop their formation or aid in treating them without surgery.

## Conclusions

The present study describes a novel vascular phenotype in which vessels form initially the retina but then traverse the ILM and anastomose with hyaloid vessels. Interestingly, these vessels are able to re-enter the retina later in development and produce properly placed retinal capillary networks (Figure 1). The data presented in this report clearly demonstrates the importance of a fully functional ILM to retinal vascular development. The present study strongly suggests that mutations in *LAMA1 *or other ILM components in humans could cause retinal diseases such as PFV, PVR, and retinal detachment. Indeed, some *Lama1^Δ ^*mutants experience retinal detachments. Finally, the observation that astrocyte ensheathment of hyaloid vessels prior to their normal regression indicates that these glial cells may contribute to the persistence of these vessels. These models could increase understanding and aid in finding treatments for PFV and PVR.

## Methods

### Mouse Husbandry

*Lama1^nmf223 ^*mutants were bred and housed at the Johns Hopkins University and all experimental procedures were performed according to the Johns Hopkins University Animal Care and Use Committee standards. Homozygous matings were used to maintain this colony. C57BL/6J (B6) animals, the background strain of *Lama1^nmf223^*, were used as controls. Mice were maintained on a 12 hr light: dark cycle with food and water ad labium. *Lama1^Δ ^*mice were bred and housed under similar conditions at INSERM with approval of the INSERM animal care and use committee. Mice were genotyped as previously described [11]. All procedures were in compliance with the ARVO statement for the use of animals for ophthalmological and vision research.

### Fundus photography and fluorescein angiography

Fundus photographs of three adult *Lama1^nmf223 ^*and three control mice were taken using a Micron III indirect camera (*Phoenix Research Labs*). Mice were anesthetized using ketamine/Xylazine and eyes dilated with atropine. Fundus photographs were taken prior to the intraperitoneal injection of 50 μl sodium fluorescein (10%; *Altaire Pharmaceuticals*) while the retina was in focus on the Micron III. Images were taken as the retinal vasculature was filling with fluorescein and once all vessels were filled.

### Immunohistochemistry

Mice were euthanized by an overdose of ketamine/Xylazine for all tissue collection. A minimum of three control and three mutant mice at each age group were used for all immunohistochemical studies. Images representative of all animals have been presented herein. For flatmount analysis, eyes were fixed for 1 hr in 2% paraformaldehyde (PFA) prior to retinal dissection and 1 hr post fixation in 2% PFA. After washing, retinas were blocked for 6 hrs at 4°C with 5% goat serum in Tris buffered saline containing 1% Triton X-100 (TBST) prior to incubation in primary antibody (diluted in 2% serum in TBST) for 18 hrs at 4°C. Following TBST washes, fluorescent conjugated secondary antibodies were applied (diluted 1:300 in 5% normal mouse serum in TBST; *Jackson Immunoresearch*) for 3 hrs. For blood vessel labeling, FITC conjugated GS isolectin (1:200; *Invitrogen*; 132450) was applied at the same time as the secondary immunohistochemistry antibody. For cryosections, eyes were cryopreserved as previously described [39]. Eight micron sections were air dried and permeabilized with cold methanol prior to blocking in 2% goat serum in TBST containing 5% BSA. Sections were incubated in primary antibody for 2 hrs, washed, and incubated in secondary antibody along with 4', 6-diamidino-2-phenylindole (DAPI; 1:1000 *Invitrogen*, D21490) diluted in TBST for 30 min. Primary antibodies included: rabbit anti-GFAP (1:200; *Dako; *Z0334), rat anti-PDGFRα (1:500; CD140a; *R&D*, 558774), and rabbit anti-pan laminin (1:750; *Sigma*; L9393), which detects multiple laminin chains. Images were captured using a *Zeiss 510 Meta *confocal microscope in the Wilmer Imaging Core Facility.

### TEM and JB-4

Eyes for TEM and JB-4 methacrylate (*Polyscience*) analysis were fixed in 2.5% PFA/2% glutaraldehyde in 0.1 M cacodylate buffer and processed as previously described [4,39,40]. Ultrastructural analysis was performed on at least two control and two mutant eyes at each age group described. At least two animals at each age from each group were embedded for JB-4. Additional animals were not analyzed because the abnormalities noted were consistent among the different ages invested as well as observations made using immunohistochemical techniques. TEM sections stained with uranyl acetate and JB-4 sections were stained with Periodic Acid-Schiffs reagent.

## List of Abbreviations

FVV: fetal vasculature of vitreous; GFAP: glial fibrillary acidic protein; GS isolectin: *Griffonia simplicifolia *isolectin B4; ILM: internal limiting membrane; PAS: periodic acid-Schiff; PFV: persistent fetal vasculature; PDGFRα: platelet-derived growth factor receptor alpha; P: post natal day; PFA: paraformaldehyde; PVR: proliferative vitreoretinopathy; TBST: Tris buffered saline containing 1% Triton X-100; TEM: transmission electron microscopy; VHP: vasa hyaloidea propria; WT: wild type

## Authors' contributions

ME participated in designing the study, performed the experiments and drafted the manuscript. DSM constructed figures for the manuscript and helped in drafting the manuscript. RG performed electron microscopy. CH maintained and collected tissue from the *Lama1^Δ ^*mice. OL developed the *Lama1^Δ ^*mice and assisted in revising the manuscript. GL designed the study, assisted with data analysis and drafted the manuscript. All authors have read and approved the final manuscript.

## Supplementary Material

Additional file 1 **Figure S1. Ultrastructure of the P1 *Lama1^Δ ^*eyes**. (A) The ILM in the *Lama1^Δ ^*mutant mouse is thin and fragmented. (B) Astrocytes (arrows) could be observed on the vitreal side of the ILM. Scale bars indicate (A: 1 μm and B: 500 nm). Click here for file

Additional file 2 **Figure S2. *Lama1^Δ ^*retinas lack retinal vessels**. Retinal vessels, labeled with GS isolectin (green), and astrocytes, labeled with GFAP (red), extend to the periphery of the control retina (A) but are completely absent from the *Lama1^Δ ^*mutant (B) at P7. Scale bars indicate 100 μm. Click here for file
