# Supplementary figures and images for: Lama1 mutations lead to vitreoretinal blood vessel formation, persistence of fetal vasculature, and epiretinal membrane formation in mice
Source: BMC Dev Biol. 2011 Oct 14;11:60. doi: 10.1186/1471-213X-11-60 (PMC3215647; doi:10.1186/1471-213X-11-60)

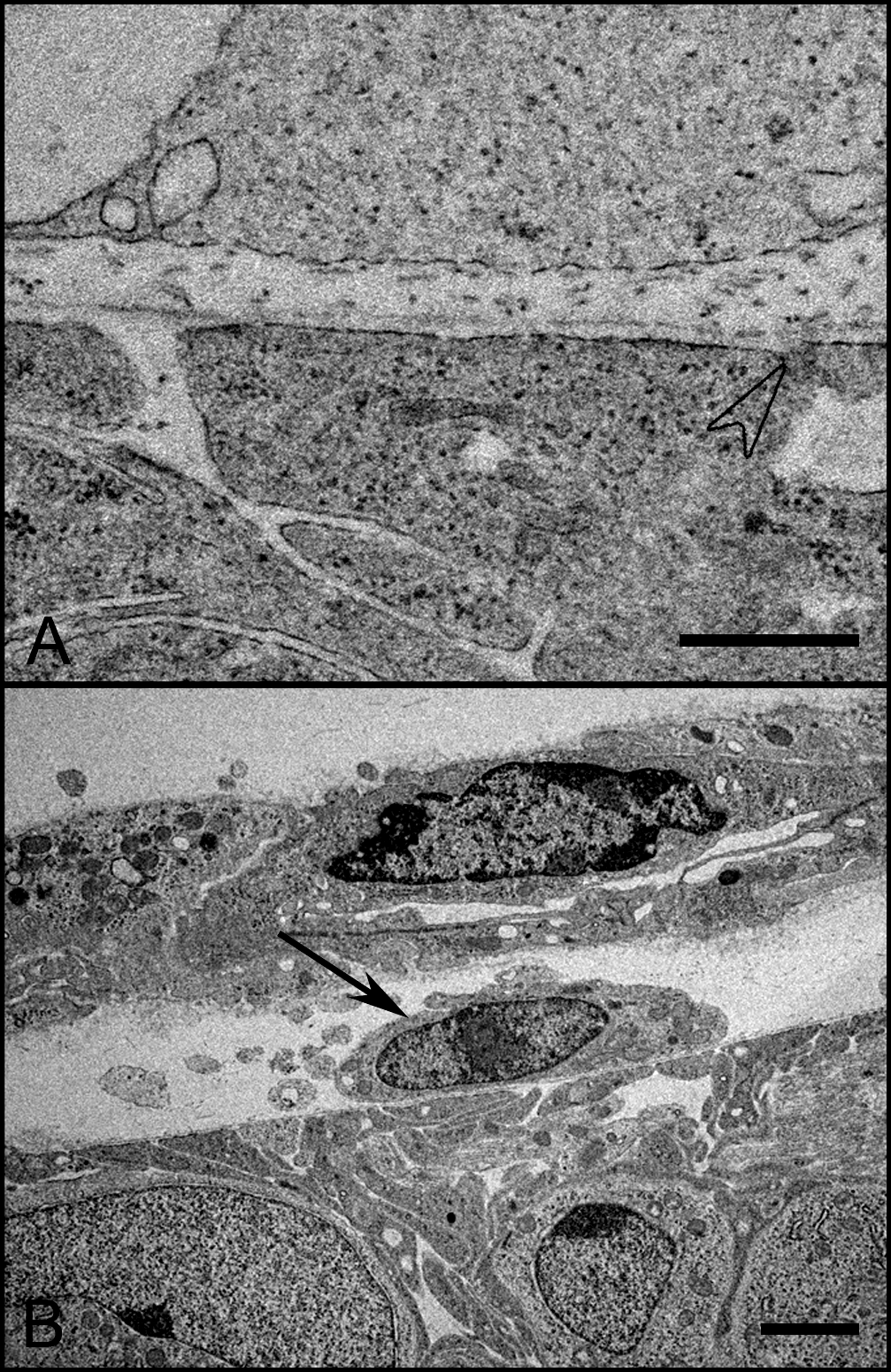

Supplement: Additional file 1 — Figure S1. Ultrastructure of the P1 Lama1Δ eyes. (A) The ILM in the Lama1Δ mutant mouse is thin and fragmented. (B) Astrocytes (arrows) could be observed on the vitreal side of the ILM. Scale bars indicate (A: 1 μm and B: 500 nm). [file 1471-213X-11-60-S1.TIFF]

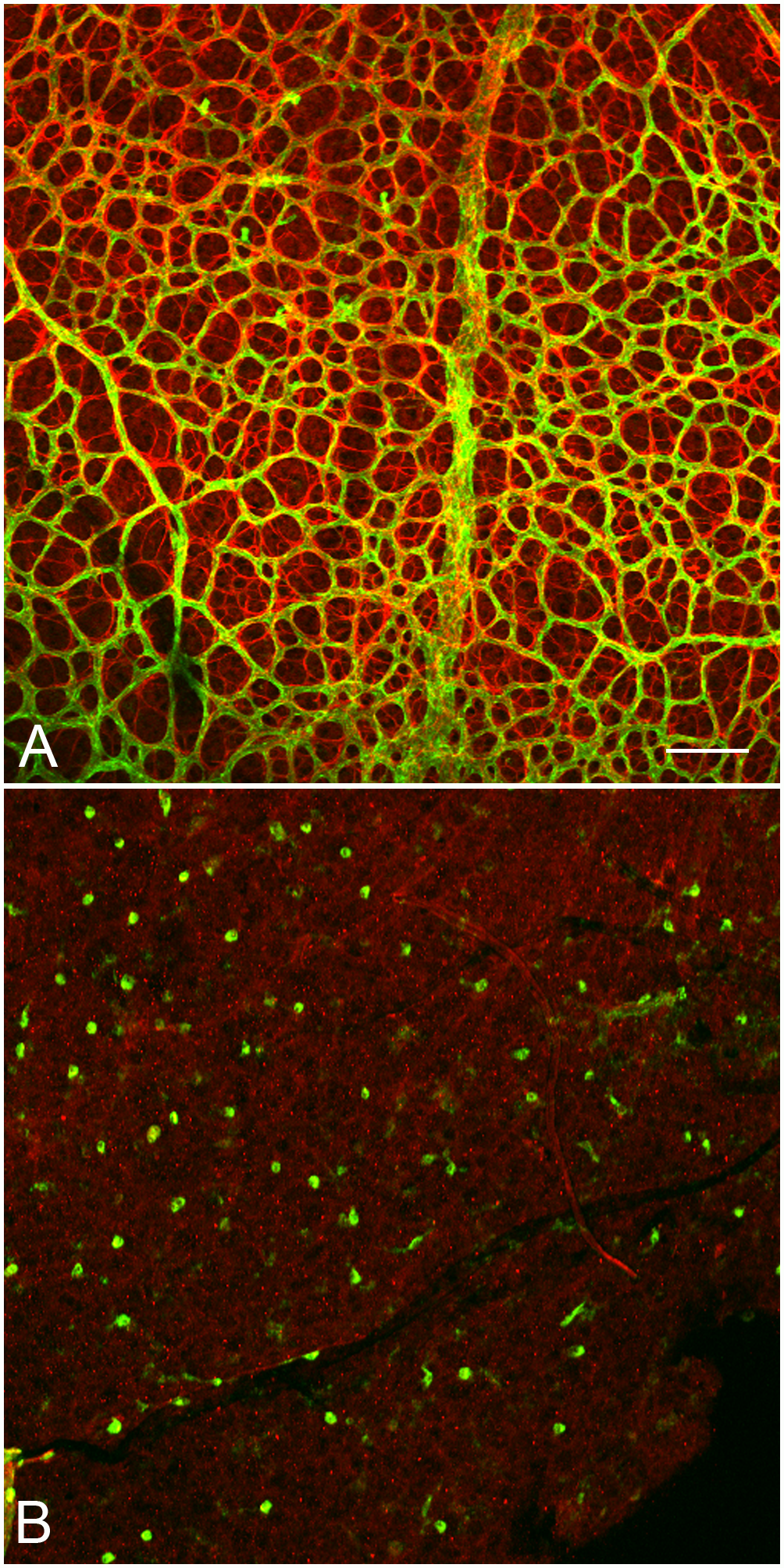

Supplement: Additional file 2 — Figure S2. Lama1Δ retinas lack retinal vessels. Retinal vessels, labeled with GS isolectin (green), and astrocytes, labeled with GFAP (red), extend to the periphery of the control retina (A) but are completely absent from the Lama1Δ mutant (B) at P7. Scale bars indicate 100 μm. [file 1471-213X-11-60-S2.TIFF]
